# Recurrent vitreous opacity caused by intraocular Toxocara larva: a case report and literature review

**DOI:** 10.1186/s12886-022-02687-2

**Published:** 2022-12-22

**Authors:** Shiqun Lin, Xiaoxu Han, Rongping Dai

**Affiliations:** Department of Ophthalmology, Peking Union Medical College Hospital, Peking Union Medical College, Chinese Academy of Medical Sciences, Beijing, 100730 China

**Keywords:** Toxocara entity, Ocular toxocariasis, Recurrent vitreous opacity, Vitrectomy

## Abstract

**Background:**

Toxocara larva entity has seldom been reported on the surface of the retina. We report on an unusual case of recurrent vitreous opacity caused by intraocular Toxocara larva after vitrectomy.

**Case presentation:**

A 34-year-old male was referred to our clinic with a 6-month history of decreased visual acuity in the right eye characterized as red, painless, and progressive. Optos fundus photograph showed optic disc elevation with granuloma, and proliferative membrane starting from the optic disc and running towards the superior temporal retina due to the movement of a Toxocara larva, which was covered by the proliferative membrane in the superior temporal retina. Since it adhered closely to the retina, the lesion in the superior temporal retina was not removed to avoid induction of an iatrogenic retinal break and the larva was not found during the first diagnostic pars plana vitrectomy. Intraocular Anti-Toxocara IgG was 45.53U (< 3, enzyme-linked immunosorbent assay (ELISA)), and the Goldmann-Witmer coefficient was 8.55, confirming the diagnosis of ocular toxocariasis. After this operation, visual acuity improved to 20/200. However, vitreous opacity worsened again, and the proliferative membrane expanded around the Toxocara larva three weeks after the operation. Toxocara larva was found and removed in the superior temporal region during the second operation. His visual acuity improved to 20/100, vitreous opacity disappeared, and the retina was stable two months after the second operation.

**Conclusion:**

It is advisable to remove suspected Toxocara larva to prevent the reoccurrence of ocular toxocariasis.

**Supplementary Information:**

The online version contains supplementary material available at 10.1186/s12886-022-02687-2.

## Background

Intraocular parasite infection has become a significant cause of blindness in many countries, especially developing countries and regions. In the proportion of intraocular parasite infection, Toxocara infection occupies the second place [1]. The migrations of larvae of Toxocara canis and Toxocara cati cause ocular toxocariasis (OT), visceral larval migration (VLM), and nervous system Toxocariasis (NT).

OT is a specific form of uveitis caused by Toxocara larvae, which enter the eyes through blood circulation due to accidental ingestion of embryonated Toxocara eggs or larvae in the tissues from domestic or wild paratenic hosts. OT mainly occurs in children. A study in the United States in 2010 showed that the average age of onset of OT was 8.5 years old, and 80.0% were less than 16 years old [2]. Woodhall et al. [3] pointed out that the average age of OT in the United States is 11.5 years old. Liu Y et al. [4] reported that the average age of onset of OT in children in China was 6 years old. Adult OT patients are comparably less, and the Toxocara entity has been seldom seen on the surface of the retina. Herein, we report a case of recurrent vitreous opacity due to the rare Toxocara larva entity.

## Case presentation

A 34-year-old male presented with a 6-month history of decreased visual acuity in the right eye characterized as red, painless, and progressive. The patient was diagnosed with "non-infectious uveitis" in another clinic and was subconjunctival injected with methylprednisolone six times, after which the condition gradually aggravated. The patient worked as a slaughterman and had five dogs.

**Fig. 1:**
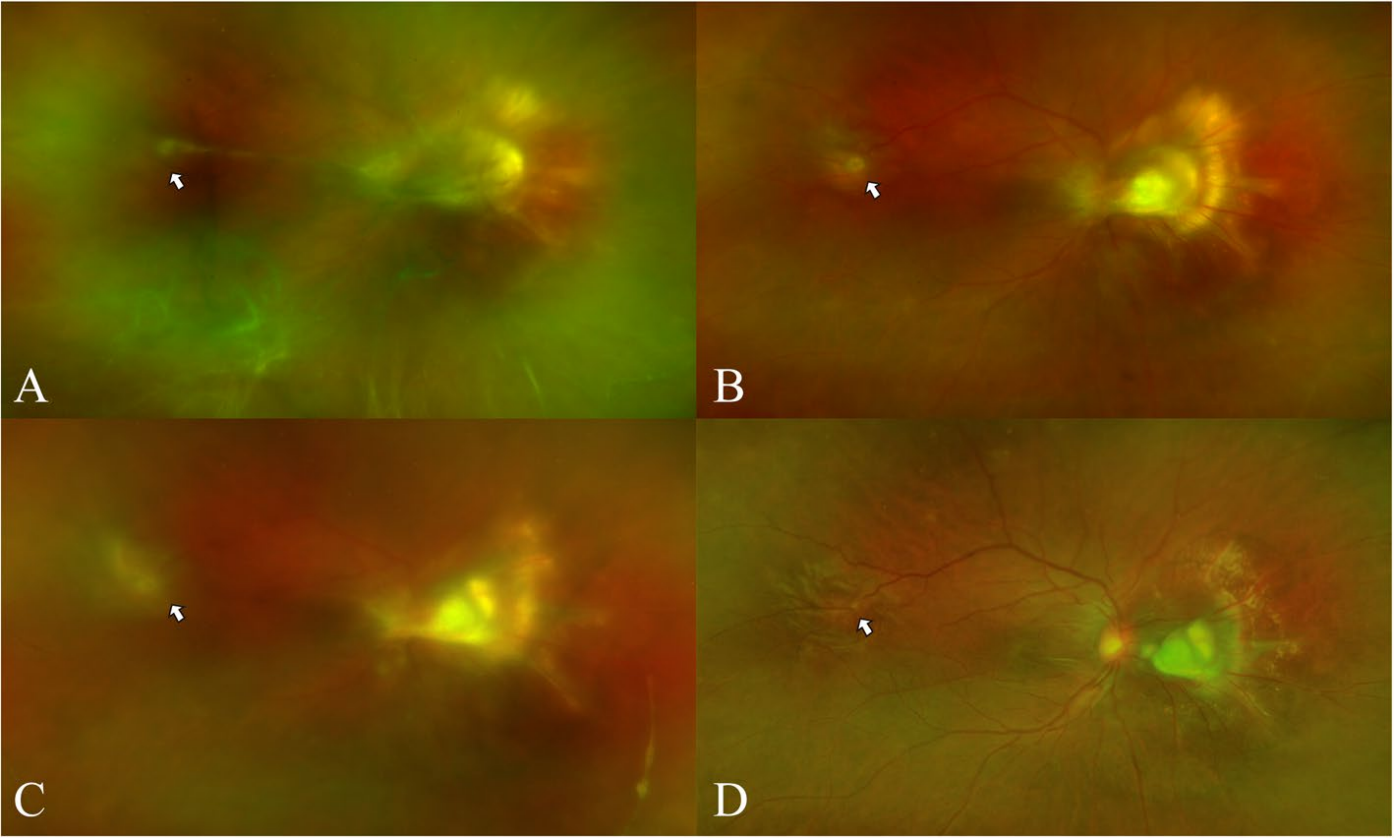
The fundus photography taken before and after operations. **A** Before the first operation, ultra-wide-field fundus photography shows vitreous inflammatory opacity, retinal granuloma and proliferative membrane in the nasal side of optic disc, and epiretinal annular yellowish lesion in the superior temporal retina. **B** One week after the first operation, the photography shows a small ring like membrane in the superior temporal retina. **C** Three weeks after the first operation, the image shows relapse of vitreous opacity and enlarged membrane in the superior temporal retina three weeks after the first operation. **D** One month after the second operation, vitreous opacity disappeared, and the retina was stable

**Fig. 2:**
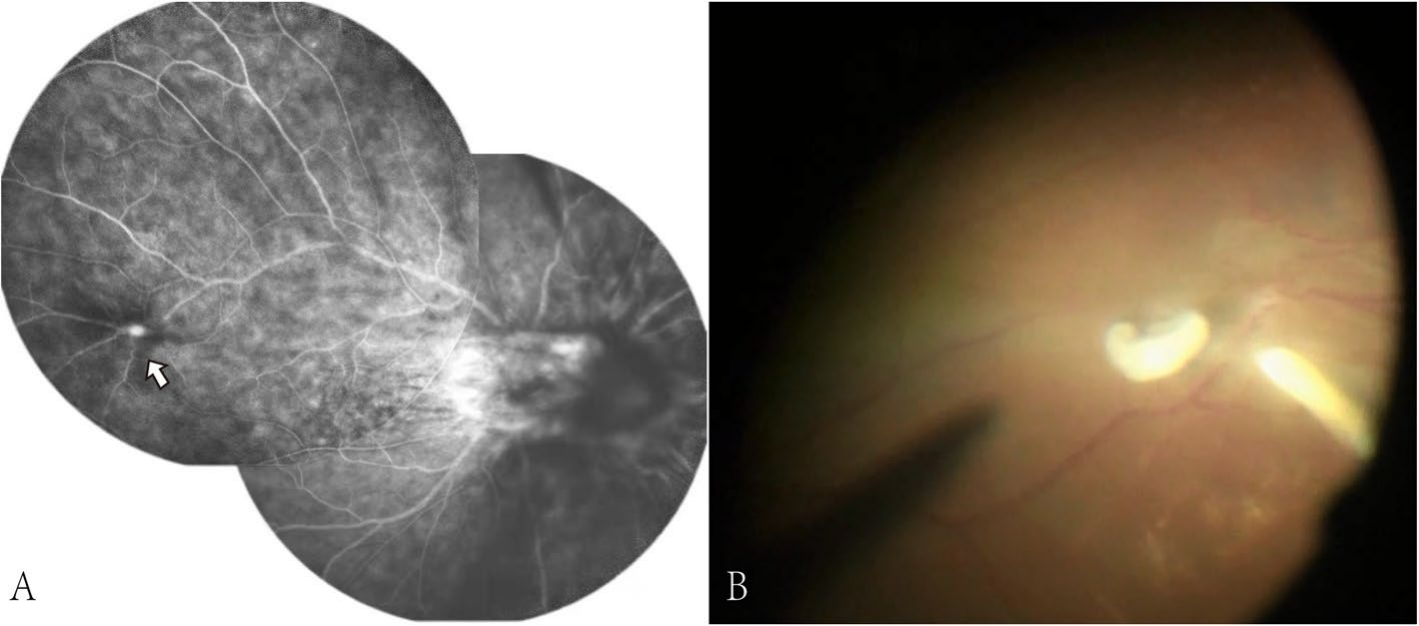
**A** The fundus fluorescein angiography showed a hyperfluorescent optic disc and two dye leakages at two corresponding retina lesions. **B** The ring-like parasite was found during the operation in the superior temporal region

**Fig. 3:**
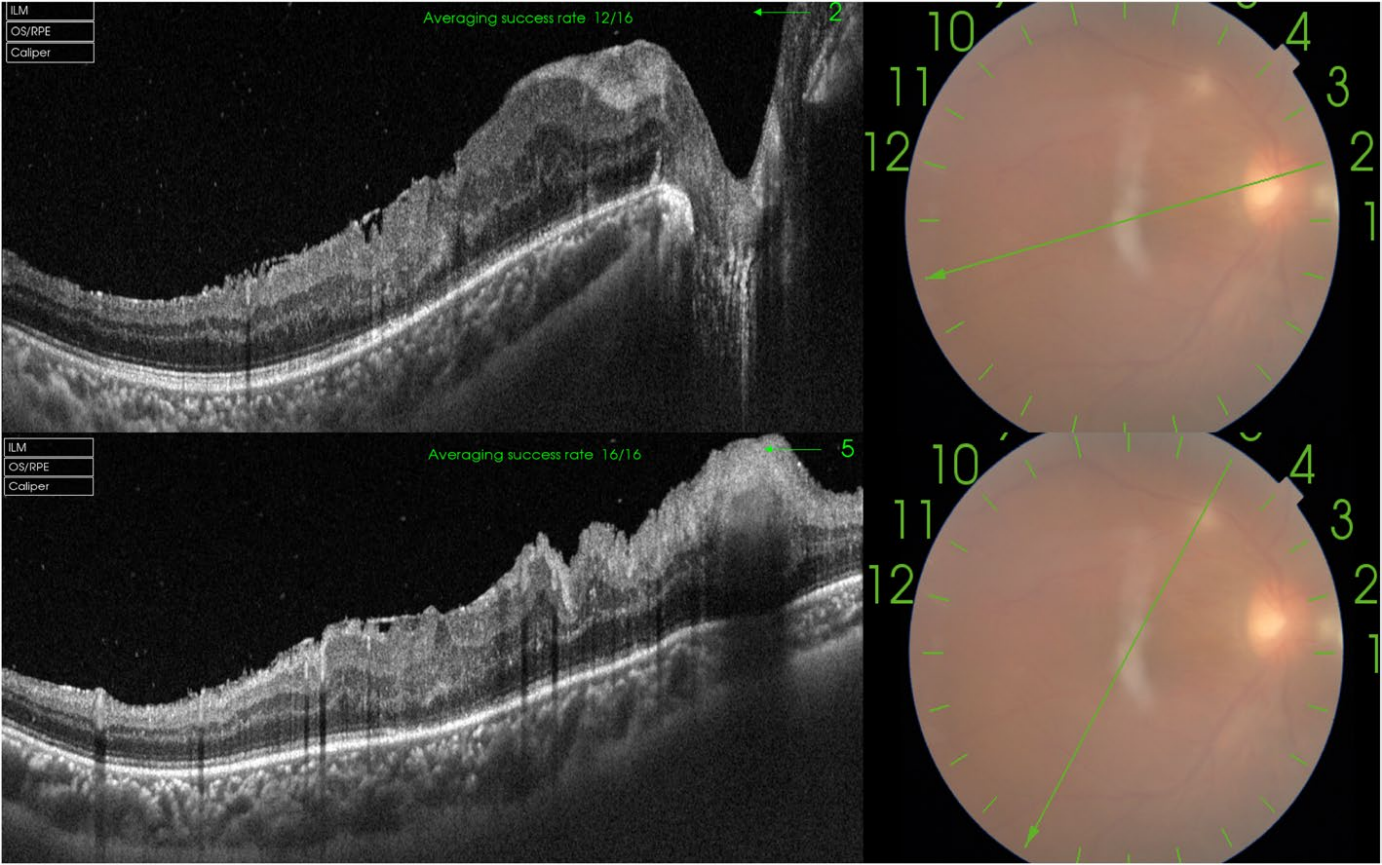
Optical coherence tomography (OCT) after the second operation. **A** Two months after vitrectomy and epiretinal membrane removal, the inner retinal layers show severe distortion and structural damage has not completely resolved. **B** There is presence of hyper-reflective masses in the inner retinal layers with posterior shadowing. In addition, the outter retinal layers show severe disorganization and photoreceptor disruption in the macular area

On the first examination, his best corrected visual acuity was 20/800 in the right eye and 20/20 in the left eye. Intraocular pressure was 18 mmHg in the right eye and 19 mmHg in the left eye. No inflammation in the anterior segment was observed. Fundus examination revealed vitreous inflammatory opacity. Optos fundus photograph (Fig. 1A) showed optic disc elevation with granuloma, and proliferative membrane starting from the optic disc and running towards the superior temporal retina. The late-phase fundus fluorescein angiography (Fig. 2A) showed a hyperfluorescent optic disc and two dye leakages at two corresponding lesions. Results of investigations revealed a negative tuberculin skin test (5 × 5 mm), negative treponema pallidum hemagglutination assay and Venereal Disease Research Laboratory test, and negative tests for human immunodeficiency virus (HIV) infection, hepatitis B surface antigen, and anti-hepatitis C antibody test. Based on clinical signs and occupational history, the diagnosis of OT was considered. To treat it and identify the diagnosis, the diagnostic pars plana was performed. Most of the proliferative membrane was stripped in operation, yet a small part of the proliferative membrane in the superior temporal region could not be removed due to the close adhesion to the retina (Fig. 1B). Since intraocular Anti-Toxocara IgG was 45·53U (< 3, enzyme-linked immunosorbent assay (ELISA)), and the Goldmann-Witmer coefficient ((Toxocara IgG in vitreous fluid/Toxocara IgG in serum)/ (Total IgG in vitreous fluid/Total IgG in serum)) is 8·55, the diagnosis of intraocular Toxocara infection was made. After this operation, visual acuity improved to 20/250. However, vitreous opacity worsened again, and the residual proliferative membrane in the superior temporal region expanded three weeks after the operation (Fig. 1C). During the second operation(supplementary video), a ring-like Toxocara larva about 300 µm to 400 µm in diameter (Fig. 2B) was observed in the superior temporal region(Additional file 1), which is a Toxocara larva. The recurrence might be due to the undiscovered larva in the eye. After the second surgery, vitreous opacity disappeared, and the retina was stable (Fig. 1D). The patient was started on oral corticosteroids (0.5 – 1 mg/kg/day oral prednisone). There was a remarkable improvement in the inflammation over the next few days. And the vision acuity recovered to 20/200 two months after the procedure. The inner retinal layers show severe distortion and structural damage has not completely resolved in the optical coherence tomography (OCT). There is the presence of hyper-reflective foci in the inner retinal layers with posterior shadowing, and photoreceptor disruption in the macular area (Fig. 3A and B). No vitreous opacity occurred and stable visual acuity (20/200) at seven months follow-up.

## Discussion and conclusions

Most of the cases in OT are unilateral in the literature, which may be caused by the migration of a single larva into the eye [5, 6]. The clinical manifestations vary, ranging from an asymptomatic state to blindness. They mainly depend on the parasite load, the intake frequency, the sites of larval migration, and the host’s inflammatory response [7].

OT is divided into three types [3]: (1) peripheral granuloma. The lesions are located from the equator to the ora Serrata, accounting for about 44% to 60%. The visual acuity at the first presentation is mostly 20/70 to 20/200 [8–10]. Dense granulomas or snowbank-like changes in the pars plana are seen in the peripheral retina. Common signs are retrocorneal deposits, iridolenticular adhesions, strabismus, moderate to severe vitritis, vitreous cords, traction retinal detachment, and epiretinal membrane [5, 6, 11]; (2) Posterior pole granuloma. The lesions in the posterior pole account for about 25% to 50%. The visual acuity at the first diagnosis is mostly 20/50 to 20/200 [8–10]. The granulomas are mostly raised on the retinal pigment epithelium (RPE) layer, and the granulomas in the macular area can cause severe damage to visual acuity [5, 12]. Common signs are moderate to severe vitreitis, traction retinal detachment, and epiretinal membrane [9, 11]. (3) Nematode endophthalmitis. About 5% to 30%. The visual acuity at presentation is mostly less than 20/200 [8, 9]. The average onset age is two years old, which is easily confused with retinoblastoma (RB) [13]. Common signs are anterior chamber reaction, severe vitritis, secondary cataract, vitreomacular traction, and epiretinal membrane [11, 14, 15]. The primary reason for visits in patients with OT is decreased visual acuity [6], mainly caused by vitreous inflammation, cystoid macular edema, traction retinal detachment, epiretinal membrane, and cataract [16].

OT is diagnosed clinically by identifying clinical signs and imaging findings combining ELISA for detecting serum and ocular fluid antibodies against the Toxocara larvae [11]. Fundus photography can detect localized lesions early, and ultra-wide-field fundus photography or ultrasound biomicroscopy (UBM) can help to detect peripheral lesions. In cases of nematode endophthalmitis in which fundus examination is not possible due to vitreous opacity, specific ancillary tests such as ultrasonography (depiction of highly reflective mass with or without vitreous band) can be helpful for differential diagnosis [7, 8]. Another feature of OT is combined (both posterior pole and peripheral granulomas) granuloma which develops as a result of the inflammatory effects in different retinal regions due to the larvae migration. Ahn et al. [10] divided it into two categories: discontinuous migration (4.3%) and continuous migration (12.9%). The former refers to long-distance migration; The latter means that the Toxocara granuloma migrates but remains adjacent to the originally observed location. If the larvae originally in the periphery travel to the posterior pole, a new lesion will occur at the posterior pole. And if it happens to be in the fovea of the macula, the vision will be severely damaged. Apart from clinical presentation, the Goldmann-Witmer coefficient ((Toxocara IgG in vitreous fluid/Toxocara IgG in serum)/ (Total IgG in vitreous fluid/Total IgG in serum)) is another critical indicator for diagnosis, whose normal range is 0–2.

Treatment for OT is mainly to reduce the inflammatory reaction in the eye and prevent the formation of proliferative membranes [6]. Glucocorticoid is a common drug to reduce inflammation and can be used alone or in combination with anthelmintics [5, 17]. Prednisone acetate eye drops can be used for patients with mild inflammation, and mydriatic drugs can be added to those with anterior segment lesions [5]. The best timing for surgery in patients with OT is before the death of the Ascaris or the formation of a significant inflammatory response [11]. Common surgical indications are persistent vitreous opacity, traction retinal detachment, epiretinal membrane, and vitreous hemorrhage.

In the reported case, the lesions in the Opotos fundus photograph and the late-phase fundus fluorescein angiography were caused by the movement of a Toxocara larva, indicating discontinuous migration (the granulomas are relocated far from the original observed location) in the eye, which is unusual in the literature. The worm migrated through retinal blood vessels, not between retinal layers. It entered the eye through the choroidal artery. And it crawled out where the small retinal artery is thin and formed a local inflammatory effect, but it did not die, so it continued to penetrate through the retinal artery and then moved to the superior temporal retina. When the retinal artery was thin, it got out again, reached the retinal surface, and then died. In the Optos fundus photograph, there was a straight band starting from the optic disc and running towards the superior temporal retina, which formed a stretch. If the larva migrated between retinal layers, the band would not be straight and there would be trace evidence. Ocular larva migrans syndrome (OLM) is characterized by an eosinophilic immune response to larval migration into the eye. After the formation of an eosinophilic abscess, a granulomatous inflammatory reaction surrounds the larvae [18]. But an encapsulated larva may not be the end. As encapsulated larvae, they remain viable for up to 10 years [19–22]. It should be noted that dormant Toxocara larvae may be reactivated at any time and migrate again [23]. Two cases of in the literature [24, 25] and our case may reveal the possible reason for discontinuous migration: the stimulation of treatment awakens the dormant larva, which all had a history of interventions before the granulomas relocated far from the original observed location.

It is worth discussing when to choose surgery. For the treatment of macular epiretinal membrane, traction and rhegmatogenous retinal detachment, vitreous surgery is fully acceptable. After all, surgery is the only way to remove the vitreous traction membrane and the epiretinal membrane, make the retina loose and reset, or remove the obstacles on the macular surface to improve vision. However, there are doubts about whether moderate or severe vitreous opacity requires surgical treatment. The possibility exists that the operation will result in the occurrence of ocular complications, which may cause more concerns for children. Therefore, whether to perform surgery requires careful evaluation of the risk/benefit ratio. For moderate or severe vitreous opacity, especially in children with hormone dependence, it is better to take vitreous surgery.

In our case, the patient was subconjunctival injected with methylprednisolone 6 times, after which the condition was gradually aggravated. Therefore, the first surgery was considered for obtaining vitreous specimens and the removal of intravitreal antigens as well as inflammatory mediators to control inflammation and improve vision rapidly. The detection of eosinophils and antibodies in the vitreous fluid is helpful for diagnosis. Vitrectomy can also reduce the recurrence of pathological changes, thus reducing local complications caused by long-term use of hormones, such as cataracts, glaucoma, and systemic adverse reactions, which is beneficial especially for children.

Our case is the first documented case of a visualized Toxocara larva with pictures and operation video. It is why at the first operation, we didn’t consider the possibility of a larva body and clear the small part of the proliferative membrane in the superior temporal region due to the close adhesion to the retina. Unexpectedly, the Toxocara larva entity was just under the small residual proliferative membrane. It is advisable to expose all proliferative membranes including that adhered closely to the retina, in case of the reoccurrence of ocular inflammation due to undiscovered Toxocara larva.

## Supplementary Information

**Additional file 1.**

## Data Availability

The datasets used and/or analyzed during the current case report are available from the corresponding author on reasonable request.

## Abbreviations

OT: Ocular toxocariasis
Ig G: Immunoglobulin G
